# A Potential Role for Substance P in West Nile Virus Neuropathogenesis

**DOI:** 10.3390/v14091961

**Published:** 2022-09-04

**Authors:** Shannon E. Ronca, Sarah M. Gunter, Rebecca Berry Kairis, Allison Lino, Jonathan Romero, Robia G. Pautler, Alan Nimmo, Kristy O. Murray

**Affiliations:** 1 Division of Tropical Medicine, Department of Pediatrics, National School of Tropical Medicine, Baylor College of Medicine and Texas Children’s Hospital, Houston, TX 77030, USA; 2 Department of Molecular Physiology and Biophysics, Baylor College of Medicine, 1 Baylor Plaza, Houston, TX 77030, USA; 3 Centre for Molecular Therapeutics and College of Medicine and Dentistry, James Cook University, Cairns, QLD 4878, Australia

**Keywords:** West Nile virus, substance P, neurokinin-1 receptor, neuroinflammation, West Nile encephalitis

## Abstract

Of individuals who develop West Nile neuroinvasive disease (WNND), ~10% will die and >40% will develop long-term complications. Current treatment recommendations solely focus on supportive care; therefore, we urgently need to identify novel and effective therapeutic options. We observed a correlation between substance P (SP), a key player in neuroinflammation, and its receptor Neurokinin-1 (NK1R). Our study in a wild-type BL6 mouse model found that SP is upregulated in the brain during infection, which correlated with neuroinvasion and damage to the blood–brain barrier. Blocking the SP/NK1R interaction beginning at disease onset modestly improved survival and prolonged time to death in a small pilot study. Although SP is significantly increased in the brain of untreated WNND mice when compared to mock-infected animals, levels of WNV are unchanged, indicating that SP likely does not play a role in viral replication but may mediate the immune response to infection. Additional studies are necessary to define if SP plays a mechanistic role or if it represents other mechanistic pathways.

## 1. Introduction

Since West Nile virus (WNV) was first detected in the US in 1999, more than 52,000 clinically evident human cases have been reported to the CDC, with approximately 2400 associated deaths. This represents only a fraction of the true disease burden, as 80% of cases are asymptomatic [1,2,3]. In fact, our previous study estimates that approximately 7 million people in the United States had been infected through 2016 [1]. Symptomatic infections typically present with a combination of symptoms, including fever, fatigue, malaise, lymphadenopathy, periocular pain, myalgia, headache, maculopapular rash, and gastrointestinal symptoms, such as nausea, vomiting, and abdominal pain [4,5,6]. Due to these nonspecific clinical manifestations of WNV infection, mild cases are often undiagnosed or misdiagnosed [7,8]. Of those infected, a small percentage will progress to neuroinvasive disease (WNND), with clinical presentations of meningitis, encephalitis, and/or acute flaccid paralysis [2]. Approximately 10% of WNND cases die. In cases of encephalitis, pathology of brain tissue reveals inflammation, which is mostly mononuclear with formed microglial nodules and perivascular clusters in white and gray matter [9,10]. Among encephalitis cases, approximately 86% experience long-term complications that decrease quality of life [4].

Substance P (SP), encoded by *TAC1*, is a member of the tachykinins distributed throughout the central nervous system (CNS) and peripheral nervous system (PNS). It is produced by most cell types, and its structure is similar in all mammalian species [11], allowing us to use mice as a valid translational model. SP is a known neuropeptide implicated in classical and neurogenic inflammation, depression, pain perception, immune modulation, and increased permeability of the blood–brain barrier (BBB) [12,13,14,15]. In conjunction with its receptor, neurokinin-1 (NK1R), SP activates different pathways depending on the cell type involved [11,12,13,16,17,18,19,20,21,22,23]. Common pathways include calcium mobilization, MAPK activation, and NFκB activation. These pathways often affect neuroinflammation and cell survival, with data to suggest that increased SP levels are associated with an exacerbated inflammatory process in the CNS during certain infections [24,25], promoting further cellular damage. In fact, studies of simian immunodeficiency virus (SIV) in macaques have identified the importance of NK1R on monocytes/macrophages, where SP and NK1R were expressed more in SIV lesions [26]. These findings could be crucial to delineate the role of SP during WNND.

In this study, we examine the potential link between WNND, SP, and the immune response to improve our understanding of disease pathogenesis. Knowing SP plays vital roles in neuroinflammatory pathways and that drug candidates are available that inhibit the neuorikinin-1 receptor (NK1R) and substance P (SP) signaling, we studied the role of SP in WNV infection by characterizing the levels of SP over the course of infection and evaluating the therapeutic effects of an NK1R inhibitor in a pilot study. We hypothesized that levels of SP would correlate with neuroinvasion and that mediation of SP would improve survival outcomes in a mouse model of encephalitic WNV disease.

## 2. Materials and Methods

### 2.1. Animals, Husbandry Practices, Monitoring for All Animals, and Specimen Collection

Female C57BL/6 (BL6) wild-type mice aged 4–6 weeks were used for these studies. Throughout the study, all animals were weighed at least once weekly and monitored twice daily for signs of illness (i.e., weight loss, unthrifty appearance, hunched up, ability to climb). Animals were humanely euthanized if weight loss exceeded 20%, and blood was collected through exsanguination under anesthesia. At necropsy, brain, whole blood, and serum were collected. Whole blood samples were stored in a 1:1 ratio in 2X DNA/RNA Shield (Zymo Research, Irvine, CA, USA) for inactivation from the BSL3. All animals were housed in an ABSL-3 laboratory. These studies were approved by Baylor College of Medicine’s IACUC committee.

### 2.2. Viruses and Drug Compounds

The WNV NY-99 strain, isolated from a crow in New York in 1999, is highly pathogenic and neuroinvasive. In wild-type BL6 mice, it typically results in lethal encephalitis. In our treatment and infected control groups, we administered this strain intraperitoneally at a dose of 10^5^ plaque-forming units. The inhibitor chosen (CH123001) is a clinical agent specifically developed as a long-term treatment option to antagonize NK1R. CH123001 is a clinical agent that was developed by Hoffmann-La Roche Ltd. (Basel, Switzerland) and is now licensed by CH Biotech Pty Ltd. (Sydney, Australia) [27]. Through a collaborative drug discovery program with James Cook University in Australia, this drug candidate was evaluated for safety, drug–drug interactions, and the specificity of the drug for its target molecule. The drug was developed to interact with the human NK1 receptor, resulting in the drug having a significantly higher affinity for the human receptor, while still being effective in rodents for preclinical testing. The full Phase I program included single (up to 800 mg) and multiple doses (up to 400 mg), pharmacodynamics, PET scan studies after single and repeated doses to evaluate brain penetration, and a cardiac safety study. The Phase II safety data trial was completed on 900 patients with daily doses of 400 mg per day for 8 weeks with no adverse events reported. Due to its high affinity and specificity for NK1R and proven safety record, we chose CH123001 to evaluate the effectiveness of treatment for WNND.

### 2.3. Experimental Design and Groups

To characterize the SP response over time during infection, mice were divided into two groups: WNV-infected and mock-infected (120 per group), with 10 mice per group per day euthanized to evaluate the time course of SP RNA and protein expression in the brain and peripheral whole blood. To evaluate the success of the NK1 inhibitor in improving outcomes, 10 mg/kg was used due to that dose’s success in alleviating injury due to traumatic brain injury in a mouse model [17]. To increase the relevance of treatment to a human infection, all doses were administered at the time of observable symptom onset (unthrifty appearance, hunched up, and/or weight loss) occurring 4–6 days post infection (DPI). Doses tested were: (1) a single dose of 10mg/kg intraperitoneally at the time of symptom onset (*n* = 5) and (2) a daily 10mg/kg IP at the start of symptom onset (*n* = 10). As controls, we had infected mock-treated mice (*n* = 8) and uninfected normal age/litter controls (*n* = 8).

### 2.4. Detection of SP and WNV

RNA was extracted from whole blood and homogenized brain tissue using the ZR Viral RNA kit (Zymo Research, Irvine, CA, USA) per the manufacturer’s protocol. SP RNA was detected using the PrimePCR assay (Bio-Rad Laboratories, Inc., Hercules, CA, USA) using manufacturer’s instructions. SP RNA levels were quantified as RNA copy numbers per µL of extracted RNA against the kit’s standard curve. WNV RNA was detected by real-time RT-PCR using TaqMan Fast Virus 1-Step Master Mix with an in-house developed TaqMan Assay [28]. A Ct value <38.5 was considered positive. As a control to validate all PCR reactions, actin amplification was evaluated. SP protein was detected in homogenized brain tissue using the SP ELISA kit (Enzo Life Sciences, Farmingdale, NY, USA) per the manufacturer’s protocol.

### 2.5. MRI

We sought to visualize BBB permeability at different timepoints of infection using a gadolinium (Gd3+) compound as imaged through Contrast-Enhanced Magnetic Resonance Imaging (CE-MRI). Up to 30 min prior to euthanasia, mice were injected with 6.4 mmol/kg of a gadolinium compound to visualize this permeability. Increased uptake, and therefore more intense color, indicated damage to the BBB, as the compound will not otherwise cross the BBB. CE-MRI experiments were performed on a horizontal bore 9.4 T Bruker Avance III, 20 cm bore Biospec Spectrometer (Karlsruhe, Germany) with a micro-imaging gradient capable of generating gradients of (1000 mT/m). A series of images was acquired over time during and after the arrival of the contrast agent in the tissue of interest. A 3D-spoiled gradient recalled echo (SPGRE) pulse sequence with a fixed flip angle of 16 degrees was used over 175 measurements with a TR = 6 ms, TE = 1.93 ms, flip angle = 16 degrees, effective bandwidth = 98.68 kHz, FOV = 35 mm × 35 mm × 20 mm with an effective partition thickness of 1.25 mm, and a matrix = 128 × 128.

### 2.6. Analysis

Descriptive statistics and two-way ANOVA were used to make comparisons of SP RNA and protein levels between infected and mock-infected mice throughout the course of WNV infection, while one-way ANOVA was used to make comparisons between the treatment groups and control groups at individual time points. Kaplan–Meier survival curves were constructed to evaluate survival and time to death, and we used the Mantel–Haenszel log-rank test to examine statistical differences between experimental groups using chi-square statistic, with an alpha of 0.05. BBB permeability was compared between groups using a two-way ANOVA and applying Tukey’s multiple comparison’s test. Survival analysis was completed using NCSS 10 (NCSS, Inc., Kayesville, UT, USA), while all other analyses and figures were completed using GraphPad Prism 8 (GraphPad Software, San Diego, CA, USA).

## 3. Results

### 3.1. SP Becomes Significantly Elevated over the Course of WNV Infection

**Figure 1:**
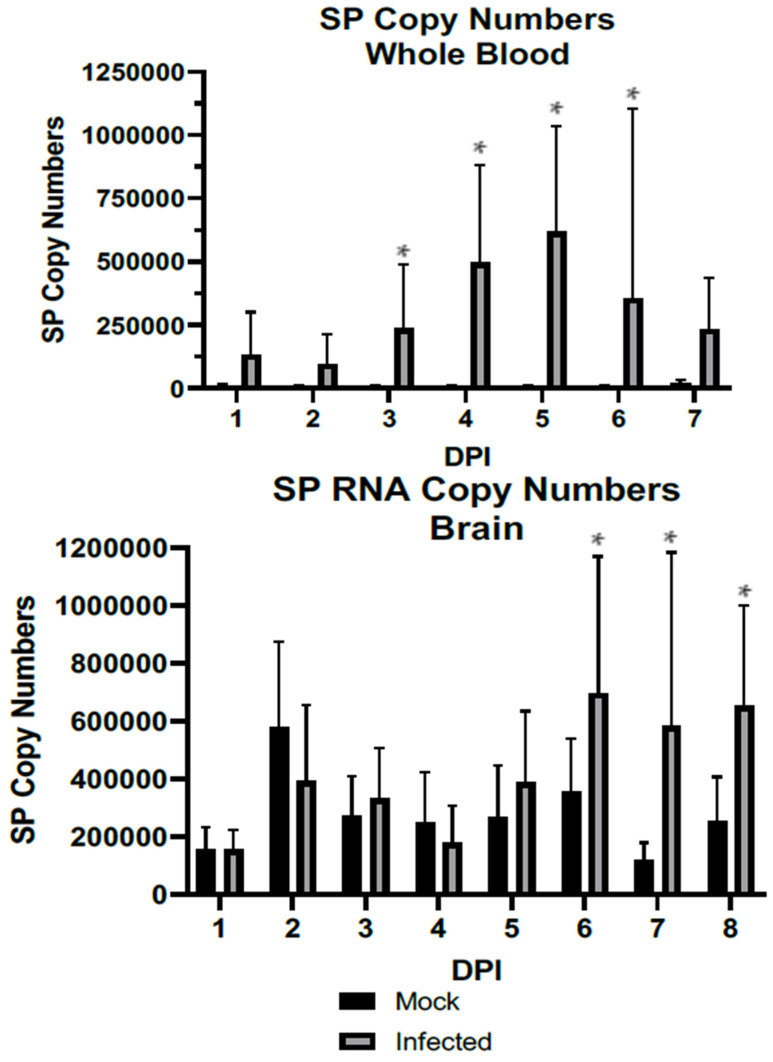
RNA levels of SP as detected by qPCR against a standard curve in whole blood and brain. **Top**: Whole blood. **Bottom**: Whole brain tissue homogenate. Error bars represent the standard deviations. Significant differences between WNV-infected and mock mice were detected within 3 DPI (*p* = 0.0092) and remained significant at 4 DPI (*p* = 0.0007), 5 DPI (*p* = 0.0009), and 6 DPI (0.0012), while SP copy numbers’ increases in the brain are not detectable until 6 DPI (*p* = 0.0031). N = 10 per group per day, and N = 18 WNV-infected mice are included in the analyses of D6 and D7 due to early attrition. *: *p* < 0.01.

When comparing WNV-infected and mock-infected mice, increased RNA transcripts are detectable in both whole blood and brains of infected mice (Figure 1). Whole blood samples were evaluated at 1–7 DPI, while brains were evaluated at 1–8 DPI to account for the longer period for infection and outcomes to reach the CNS. An increase is first detectable in the whole blood, is significant within 3 days of infection (DPI) (*p* = 0.0092), and remains significant at 4 DPI (*p* = 0.0007), 5 DPI (*p* = 0.0009), and 6 DPI (0.0012), while the SP copy numbers’ increase in the brain is not detectable until 6 DPI (*p* = 0.0031), which correlates to the onset of signs consistent with disease (weight loss, hunching, etc.). SP RNA levels in the brain remain significantly elevated in the WNV-infected group at 7 and 8 DPI (*p* = 0.0037 and 0.0022, respectively), at which time the study concluded.

**Figure 2:**
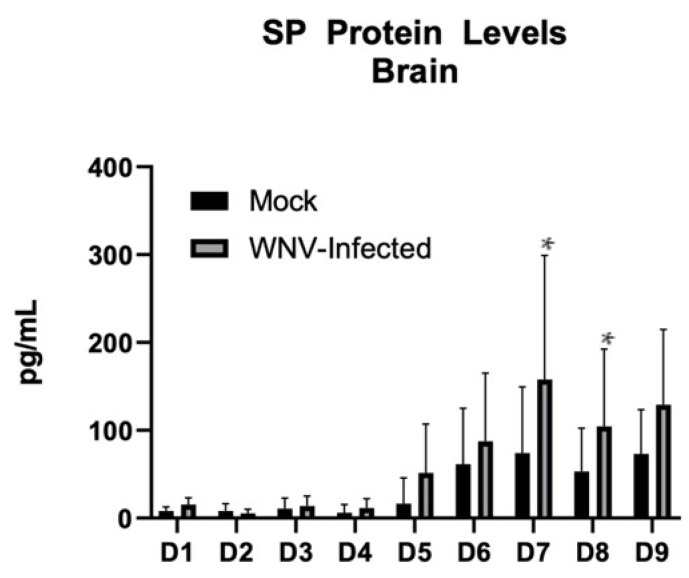
SP as detected by ELISA against a standard curve in whole brain homogenate. Error bars represent the standard deviations. Significantly higher mean protein levels were detected in WNV-infected mice when compared to mock at both 7 DPI (*p* = 0.017) and 8 DPI (*p* = 0.047). N = 10 per group per day, and N = 18 WNV-infected mice are included in the analyses of D6 and D7 due to early attrition. *: *p* < 0.05.

To be certain that the increased levels of RNA were biologically relevant, protein levels of SP were evaluated in the brain (Figure 2). Protein levels began to increase in both WNV-infected and mock-infected controls at 5 DPI, and there were significantly higher mean protein levels detected at both 7 DPI (*p* = 0.017) and 8 DPI (*p* = 0.047) in the WNV-infected when compared to mock-infected mice by two-way ANOVA. Protein levels in whole blood were not evaluated.

### 3.2. Blood–Brain Barrier Permeability Is Visualized during Infection

**Figure 3:**
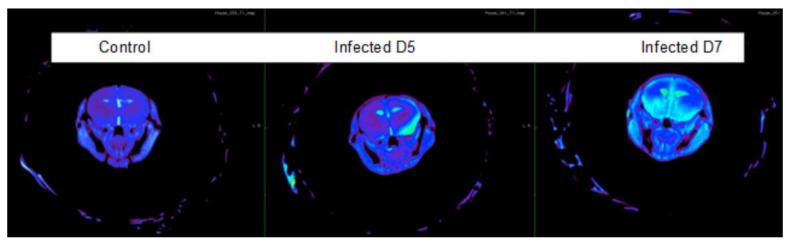
Blood–brain barrier permeability over time. These images represent one control mouse unexposed to WNV at 5 DPI and two mice infected with WNV at 5 and 7 DPI. An increase in color is indicative of higher signal intensity.

By evaluating images obtained through a preliminary CE-MRI analysis, it was apparent that permeability moved from the right to the left hemispheres, with differences between controls and WNV-infected mice detected at 5 DPI (*p* = 0.043) (Figure 3) (Supplemental Table S1). Significant intensity changes were detected in the left hemisphere by 7 DPI in infected mice (*p* = 0.0026). Due to a small sample size (N = 3 per group), significance was not observed for all time points, but this highlights the need for future studies, as this observation supports that this technique is a valuable tool, but it does not suggest that this correlation is direct causation.

### 3.3. CH123001 Increased Survival in Treatment Groups and Prolonged Time to Death

**Figure 4:**
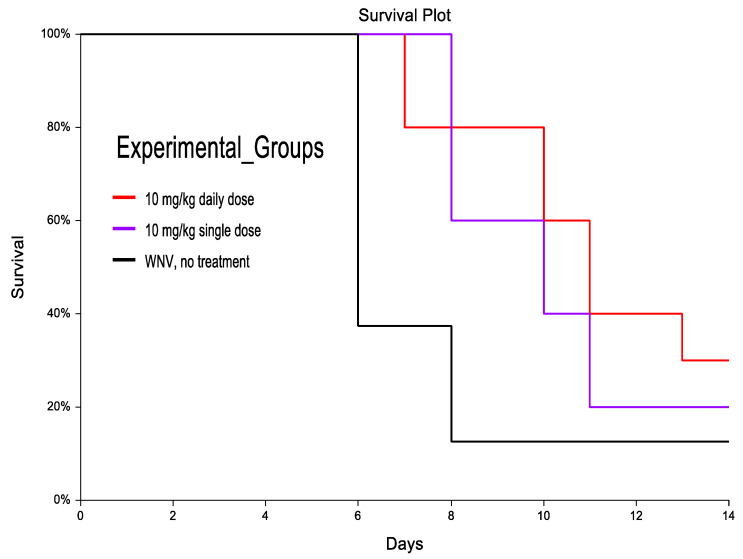
Kaplan–Meier analysis in treated and untreated mice exposed to WNV-NY99. Red: WNV-infected + 10mg/kg daily dose beginning at symptom onset (N = 10); Purple: WNV-infected + 10 mg/kg single dose at symptom onset (N = 5); Black: WNV-infected with no treatment (N = 8).

In a pilot proof-of-concept study, in mice treated with a single dose of CH123001 at 10mg/kg at the first signs of disease onset, which occurred at 4–6 DPI, survival increased to 20% (1 out of 5) versus 12.5% (1 out of 8) in the untreated group (Figure 4). Similarly, time to death was prolonged in the single-dose treatment group (8–11 DPI) when compared to the untreated group (6–8 DPI). While there was an observable difference in survival and delay to death among the single-dose group when compared to the untreated group, the difference was not statistically significant by the Mantel–Haenszel logrank test (*χ*^2^ = 1.95; *p* = 0.16), which is possibly due to the smaller sample size of the single-dose treatment group (*n* = 5). In mice treated daily with 10mg/kg CH123001 at the first signs of disease onset, survival was further increased to 30% (3 out of 10) versus 12.5% in the untreated group. Time to death in mice treated daily was also extended to 8–13 DPI. Based on the Mantel–Haenszel logrank test, there was a significant difference in survival distribution between the WNV-infected mice treated daily with CH123001 and no treatment (30% versus 12.5%; *χ*^2^ = 4.3; *p* = 0.037). Larger sample sizes are needed to confirm this.

### 3.4. SP Levels Were Decreased in the Treated Groups and Correlated with Survival

**Figure 5:**
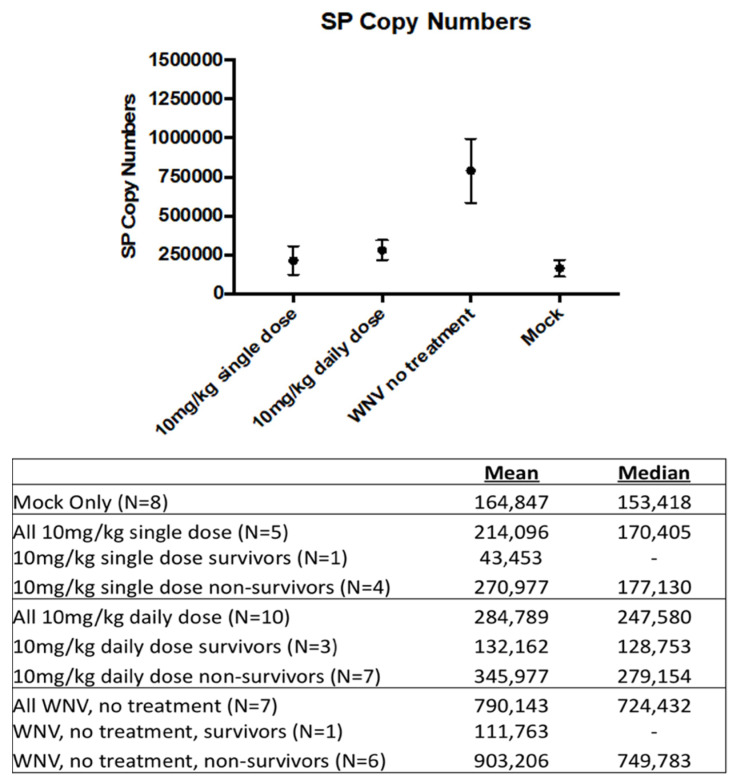
Comparison of SP levels between treatment groups at time of euthanasia. Means are expressed with the standard error. SP levels correspond to the number of SP RNA copies per µL of RNA. For these reactions, 2 µL of RNA was used.

When the levels of SP expression in brain tissue were compared between groups at time of euthanasia, a significant difference was identified between treated and untreated groups (Figure 5). There was no significant difference in SP levels between the single dose, the daily dose, or the mock groups, but SP levels were significantly different between those groups and the WNV-no treatment group (*p* < 0.05 for all).

**Figure 6:**
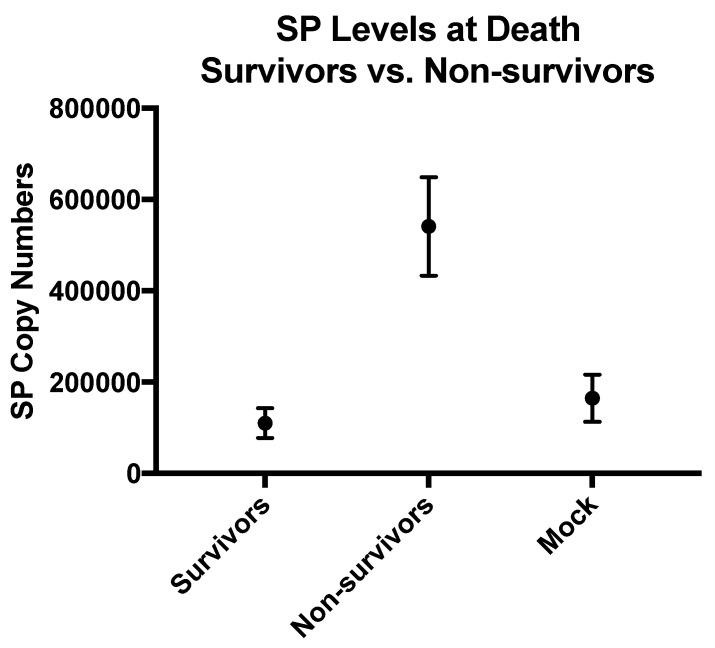
Comparison of SP levels between survivors, non-survivors, and mock-infected animals at time of euthanasia. Means are expressed with the standard error. SP levels correspond to the number of SP RNA copies per µL of RNA. For these reactions, 2 µL of RNA was used.

We then quantified the levels of SP RNA in the brain at the time of euthanasia. When we compared SP RNA levels in brain tissue between survivors and non-survivors, we found that lower levels of SP RNA copy numbers in the brain correlated with longer survival (Spearman’s ρ = −0.7463). The levels of SP were significantly lower in survivors versus non-survivors (Mann–Whitney test, *p* = 0.0026) (Figure 6).

### 3.5. Levels of WNV RNA Were Not Affected by CH123001 Treatment

**Figure 7:**
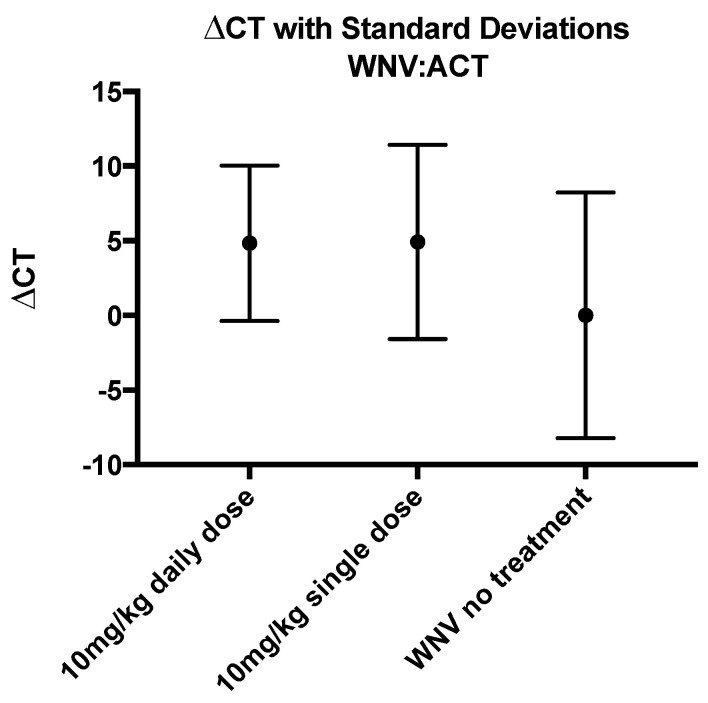
WNV amplification from whole blood at 7 DPI. Levels of WNV RNA were determined and normalized to ACT to calculate the ∆CT for each treatment group. No significant differences in the levels of WNV RNA were found between each treatment group when compared using a One-Way ANOVA.

To aid in determining if decreases in SP levels were directly related to increased survival, we evaluated the levels of WNV RNA in each group at 7 DPI, the time at which virus should be detectable in all infected animals. We used actin (ACT) RNA expression levels as an internal control and calculated the ∆∆CT of ACT to WNV. We used one-way ANOVA to evaluate the difference in the mean ratio between groups and found no significant difference (*p* = 0.0644) in WNV levels when normalized to ACT between daily dose, single dose, and untreated groups (Figure 7). This provides initial support that controlling the immune response, and not WNV replication, is crucial for increasing survival.

## 4. Discussion

Although many advancements have been made in delineating the pathogenesis of WNV, there remain no therapeutic options or preventive modalities to combat disease and improve patient outcomes. Clinical management of WNND is limited to supportive care. Preliminary accounts of effective alternative agents have been reported, including antiviral agents, immunomodulating agents, angiotensin-receptor blockers, and nucleic acid analogues [5,6,29,30], but these agents are not FDA-approved for clinical treatment of WNV, and issues with enrollment of acute cases have impacted efforts to validate the efficacy of these new drug candidates [31].

This study provides new evidence that SP plays a potential role in WNV infection in a mouse model of encephalitic infection and that additional investigation into this topic is warranted. Increased SP levels were observed at both the RNA and protein levels, and these correlated with symptom onset and criteria for human euthanasia, potentially indicating it can drive a cascade of neuroinflammatory processes previously associated with WNND. We also found that interrupting SP/NK1R signaling significantly delayed time to death and improved survival when given 10 mg/kg daily at the first sign of symptom onset. Former pathology studies of WNND brain tissue reveal an inflammatory response that impacts the permeability of the BBB during acute infection, which we visualized using MRI at the time that SP increased. Previous MRI studies of acute infection in humans indicate changes to the basal ganglia, thalamus, pons, and lobar gray and white matter [32,33,34], while MRI studies conducted years after infection indicate cortical thinning and regional atrophy throughout the brain [35]. Due to the many possible effects of SP on the brain and other data that suggest immune modulation plays a role in BBB permeability during WNV infection, it is not unreasonable to hypothesize that some of this damage could be related to SP-regulated pathways, although additional studies are required to confirm or dismiss this link.

It is important to discuss the increased expression of SP throughout the study, which was also seen in the mock-infected animals, although the statistical analyses support that there remains a significant increase in SP in infected animals when compared to mock-infected animals. SP is both abundant and widely distributed within the CNS. In addition to its proposed role in inflammatory responses in the CNS [13,17], it is also considered to play an important role in a number of higher brain functions, including anxiety and the response to stress [36]. Indeed, changes in SP levels in response to stressful stimuli have led to antagonists of SP and its NK1R being viewed as potential anxiolytic agents [37,38]. Ebner and colleagues [37] demonstrated not only the release of SP in response to stress, but also that there was a relationship between the magnitude of release and the intensity of the stressful stimulus. In a 2006 review, Ebner and Singewald summarized the evidence supporting the central release of SP in response to both emotional and physical stressors [18]. Whilst good laboratory practice aims to reduce physical and emotional stress to a minimum, it is not possible to completely avoid their impact [39]. One of the key tools to minimize any suffering or stress is the use of general anesthesia. While anesthesia represents an essential component of laboratory practice, repeated use of the general anesthetic isoflurane is considered as a mild stressor, with the impact mainly being in the post-anesthetic recovery phase [40]. Behavioral studies assessing the impact of anesthesia have demonstrated that isoflurane, and in particular repeated administration of isoflurane, can heighten anxiety responses [40,41,42]. In the current study, for the mock group of animals to serve as a valid control, it is essential that they are exposed to the same emotional and physical stimuli as the study group. Considering what is known about SP’s role in the response to stressful stimuli, it is to be expected that a rise in SP levels would be observed within the mock group in response to the handling, immobilization, and regular anesthetization using isoflurane. However, given that the control group accurately mirrors the interventions employed for the WNV-infected group, the difference in SP levels in response to infection may reflect the additional impact of the inflammatory response.

This study has its limitations. Groups sizes were small and varied for CH123001 due to limited availability of the experimental NK1R inhibitor, but we were able to detect significant differences between groups due to the drastic difference in SP levels. Administration of drugs had to be done under anesthesia, which likely explains the higher-than-expected mortality in the daily treatment groups and the increase in SP levels in mock-infected mice. This can be mitigated in the future by using an NK1R inhibitor with a longer half, such as those approved by the FDA. Future studies must use larger sample sizes to confirm the results of this study and to evaluate additional immunomodulating pathways relevant to WNND and to SP/NK1R.

The results from our study indicate that CH123001, a NK1R inhibitor, has therapeutic potential, consistent with what is known about SP/NK1R in other fields. NK1Rs are valuable targets for reversing the effects of traumatic brain injury and other neurological damage. One such NK1R inhibitor, N-acetyl-L-tryptophan (NAT), has shown promise in animal studies against inflammation and swelling following traumatic brain injury. In rats, NAT attenuated edema formation and reduced BBB permeability after brain trauma, and in mice, NAT improved motor and cognitive outcomes [17]. Neither CH123001 nor NAT are FDA-approved at this time; however, there are currently two NK1R antagonists approved for clinical use in the United States: aprepitant (Emend; Merck & Co.) and rolapitant (Varubi; Tesaro). Both aprepitant and rolapitant are approved for use to treat chemotherapy-induced nausea and vomiting, and while these drugs have primarily been studied in patients undergoing cancer treatment with cisplatin, aprepitant has also been evaluated in clinical trials to treat HIV-infected patients as an anti-inflammatory. 

While the results of the pilot treatment study suggest promising outcomes, we need to understand its mechanisms of action in the context of infection. SP, acting via its preferred receptor, the NK1 tachykinin receptor, exerts a range of biological activities, including immune modulation [21]. It is postulated that SP may represent a mediator that enables cross communication between the nervous and immune systems [43]. In terms of the CNS, it has also been demonstrated that SP has the ability to activate microglia and contribute to the development of microglia-mediated inflammation in the brain [19,23]. In addition to its range of direct biological activities [20], SP plays a role in the elaboration of inflammatory mediators [22]. Mitogen-activated protein (MAP) kinase pathways are a critical component of cell signaling and are involved in regulating a range of cell functions. SP, acting on its NK1 receptor, has been shown to activate the ERK1/2 and p38 MAP kinase pathways, leading to SP-mediated nuclear translocation of phosphorylated NF-κB, leading to selective inflammatory chemokine production [22]. This elaboration and proliferation of inflammatory mediators can result in a positive feedback situation. As SP itself regulates the expression of a range of cytokines, the SP/NK1R pathway is, in turn, regulated by inflammatory activity. A reciprocal interaction has been demonstrated between SP and the cytokines interleukin-12 and interleukin-23. SP will enhance the expression of a range of cytokines, including IL-12 and IL-23, whilst IL-12 and IL-23 will increase the expression of substances [16,44]. Hence, if SP-mediated inflammation leads to a positive feedback situation, where it increases SP expression further, it is possible to conceive that inhibition of this cycle using a selective NK1R antagonist may lead to reduced transcription of the *TAC1* gene and SP expression. Since CH123001 blocks the cellular receptor for SP, we believe this then prevents a destructive inflammatory cascade that is driven by viral pathogenesis. We hypothesize that SP is released in response to WNV infection, which then leads to cytokine-induced inflammation, thereby increasing edema within the brain, facilitating neuronal apoptosis, and damaging the integrity of the BBB. Our findings provide preliminary support for our hypothesis, but these data indicate that further studies delineating the specific mechanisms of action of CH123001 and the role for SP are necessary to determine if the increase in SP observed in this study is directly related to pathogenesis or if it is a biomarker of another pathway. Future studies will need to perform more mechanistic studies, especially using SP/NK1R knockout or knockdown models of infection, and evaluate FDA-approved NK1R inhibitors if these future studies suggest a mechanistic role for SP/NK1R in WNV infection.

## 5. Conclusions

Overall, our study provides preliminary evidence to support a role for SP during WNND. Expanding these studies will lead to an increase in understanding WNV pathogenesis by defining if SP plays a mechanistic role in infection or is a biomarker of other mechanistic pathways. Long term, this will allow us to identify potential therapeutic options that could have a significant public health impact by decreasing the risk of death, reducing the economic burden associated with WNV infection, preventing long-term complications from infection, and improving patient outcomes.

## Data Availability

Data that are not available in the manuscript can be requested from the corresponding authors.

## Supplementary Materials

The following supporting information can be downloaded at: https://www.mdpi.com/article/10.3390/v14091961/s1, Table S1: Raw Signal Intensity Data.
