# Supplementary material for: A Potential Role for Substance P in West Nile Virus Neuropathogenesis
Source: Viruses. 2022 Sep 4;14(9):1961. doi: 10.3390/v14091961 (PMC9503494; doi:10.3390/v14091961)
Supplement: Supplementary file 1 [file viruses-14-01961-s001.zip › viruses-1905645-supplementary/viruses-1905645-supplementary.pdf]

Supplemental Table S1
Raw Signal Intensity Data.

Animal	Treatment	Signal Intensity (ASU)	
		Left Hemisphere	Right Hemisphere
1	Mock, 5dpi	104	110
2	Mock, 5dpi	91.9	105
3	Mock, 5dpi	91.4	114
4	WNV, 5dpi	134	152
5	WNV, 5dpi	108	121
6	WNV, 5dpi	112	123
7	WNV, 7dpi	163	166
8	WNV, 7dpi	139	125
9	WNV, 7dpi	119	119
